# Yiqi Formula Enhances the Antitumor Effects of Erlotinib for Treatment of Triple-Negative Breast Cancer Xenografts

**DOI:** 10.1155/2014/628712

**Published:** 2014-10-19

**Authors:** Ming-juan Liao, Mei-na Ye, Rui-juan Zhou, Jia-yu Sheng, Hong-feng Chen

**Affiliations:** ^1^Laboratory of Surgery, Shanghai University of Traditional Chinese Medicine, Shanghai 200032, China; ^2^Department of Breast, Longhua Hospital, Shanghai University of Traditional Chinese Medicine, Shanghai 200032, China

## Abstract

Yiqi formula (YF), a traditional herbal prescription, has long been used to treat triple-negative breast cancer (TNBC) patients. The present study aims to investigate the effects and the related mechanism of YF for treatment of TNBC xenografts. MDA-MB-231 (human TNBC) cells were subcutaneously injected into the second mammary fat pad of 40 female nude mice, which were divided into four groups: control, erlotinib (an epidermal growth factor receptor (EGFR) tyrosine kinase inhibitor), YF, and combination (YF plus erlotinib). All treatments were administered orally for 30 days. Inhibition rate of tumor weight by erlotinib, YF, and the combination was 26.47%, 17.24%, and 39.15%, respectively. Western blotting showed that YF, erlotinib, and the combination downregulated p-EGFR (*P* < 0.01) and p-Akt1 (pT308) (*P* < 0.05) and upregulated PTEN compared with control, and the combination was more efficacious than erlotinib alone (*P* < 0.05). Similar results were detected by immunohistochemistry. Real-time quantitative PCR showed that YF, erlotinib, and the combination increased PTEN mRNA (*P* < 0.05, *P* < 0.01) compared with control, and the combination was more efficacious than erlotinib alone (*P* < 0.05). In conclusion, YF can regulate the main components of the PI3K/Akt pathway in TNBC xenografts. When YF was used in combination with erlotinib, it enhanced the antitumor effects of erlotinib on TNBC xenografts. These findings suggest that YF is suitable to use for the treatment of TNBC patients.

## 1. Introduction

Triple-negative breast cancer (TNBC) is characterized by lack of expression of estrogen receptor (ER) and progesterone receptor (PgR) and overexpression of human epidermal growth factor receptor 2 (Her-2). TNBC accounts for 12–17% of all breast cancers and shares similarities with basal-like breast cancer (BC), claudin-low BC, and BRCA1-related disease [1, 2]. The epidemiological and clinical characteristics of TNBC are distinct from those of other BC subtypes, including younger age at diagnosis, higher risk of relapse in spite of increased chemosensitivity, and a higher incidence of lung and brain metastatic relapses. Treatment options are limited because the endocrine therapies and HER-2 antagonists typically used for other BCs are ineffective; thus conventional cytotoxic chemotherapy remains the mainstay of current systemic management [3]. Combined chemotherapy is commonly used clinically and is a promising method of improving cancer treatment [4]. It has been evidenced that these tumors are highly chemosensitive, but the results remain unsatisfactory [5]; therefore management of TNBC is challenging. Complementary therapies including traditional Chinese medicine (TCM) have enjoyed a growing popularity due to their reputed safety and clinical efficacy for BCs. It is believed that TCM produces synergistic effects and a reduction of side effects and toxicity.

The phosphatidylinositol 3-kinase (PI3K)/protein kinase B (also known as AKT) signaling pathway is an important regulator of cell survival, proliferation, angiogenesis, and growth. It is frequently deregulated in a majority of human cancers through receptor tyrosine kinase activation or amplification such as loss of* PTEN* (phosphatase and tensin homolog), activating mutations of* PIK3CA*, or other downstream effectors of PI3K/Akt signaling. This pathway is activated in approximately 70% of human BCs and is implicated in resistance to endocrine therapy, chemotherapy, and radiotherapy [6, 7]. Consequently, inhibitors of PI3K/Akt signaling are currently being tested in clinical trials for BC. TNBC shows enhanced PI3K activity, mainly through loss of* PTEN* [8, 9].

A large number of herbal remedies (e.g., garlic, mistletoe, Essiac, Lingzhi (mushrooms of the genus* Ganoderma*), and* Astragalus* (*Astragalus membranaceus*; Huang Qi)) are used by patients with cancer, including BC, for treating the cancer and/or reducing the toxicities of chemotherapeutic drugs. Yiqi principle of TCM is commonly used in clinical treatment of TNBC patients. Yiqi formula consists of* Astragalus* and* Poria cocos* (Fu Ling), which are on behalf of Yiqi principle.* Astragalus* is the sovereign drug of the formula. As shown in our previous study, injection of* Astragalus* and its monomers can inhibit the proliferation of TNBC cell lines such as MDA-MB-468 and MDA-MB-231, and the antiproliferation mechanisms may be related to the regulation of key proteins in the PI3K/Akt pathway [10–13]. We also carried out experiments using Yiqi formula on TNBC cell lines.* In vitro* research also showed an inhibiting effect of YF.

In the current study, we carried out further investigation into the effects and mechanisms of YF on TNBC* in vivo*.* In vivo *cotreatment of erlotinib and YF enhanced the antitumor effects of erlotinib.

## 2. Materials and Methods

### 2.1. Animals

The study was approved by the Institutional Animal Care and Use Committee at Longhua Hospital and conducted according to the guidelines for the Care and Use of Laboratory Animals, which follows the guidelines of the National Institutes of Health.

Forty 6–8-week-old female athymic nude mice (Shanghai Laboratory Animal Center, Shanghai, China; certificate number SCXK2007-0005) were used. Mice were maintained under specific pathogen-free (SPF) conditions on a 12-h light/dark cycle and had free access to water and standard laboratory feed.

### 2.2. Cell Lines and Culture

MDA-MB-231 cells (Type Culture Collection, Chinese Academy of Sciences, Shanghai, China) were maintained in L-15 medium supplemented with 10% fetal bovine serum and 100 units/mL penicillin/streptomycin. Cells were cultured at 37°C with 5% CO_2_ in a humidified incubator and passaged with 0.05% trypsin every 2-3 days.

### 2.3. Drugs

Erlotinib (as erlotinib HCL tablets; Tarceva, Roche Pharmaceuticals) was dissolved in 0.9% NaCl solution. A water decoction of 0.5 g/mL of YF (ratio of* Astragalus* :* Poria* 24.5 g : 14.5 g) was obtained from our Pharmaceutical Preparation Department (Longhua Hospital, Shanghai University of Traditional Chinese Medicine, Shanghai, China). Based on an equivalent dose conversion for mice, the dosage given was 4.75 g/kg YF and 18.75 mg/kg erlotinib. Treatments were given once daily at this dosage and administered orally by gavage, daily at the relevant dosage. Control mice received the corresponding vehicle (0.9% NaCl or water). Reference standards of calycosin glycosidase, astragaloside, and pachymic acid were purchased from the Chinese Academy of Food and Drug Testing.

### 2.4. High Performance Liquid Chromatography

We used high performance liquid chromatography (HPLC) for simple chemical composition analysis of YF. A 2 mL sample of YF was ultrasonically extracted with 8 mL 70% methanol for 30 min. The supernatant was collected, and the solution was filtered through a 0.22 *μ*m nylon-membrane filter (Millipore, Barcelona, Spain) prior to injection into the HPLC system (Agilent Series 1200; Agilent Technologies, USA), which consisted of a dual pump, an autosampler, an evaporative light scattering detector (ELSD) (Alltech Associates, Deerfield, IL, USA), and a column compartment that used HP ChemStation Software. The column temperature was maintained at a constant 30°C, and the mobile phase flow rate was 1.0 mL/min. The mobile phase consisted of acetonitrile (solvent A) and 0.1% formic acid aqueous solution (solvent B), and the gradient elution was optimized as follows: 0–30 min, 20–40% A; 30–60 min, 40–60% A. The analytes were monitored at 260 nm.

### 2.5. Establishment of Xenografts

MDA-MB-231 cells (2 × 10^6^) were resuspended in 0.1 mL PBS and injected subcutaneously into the second mammary fat pad of each mouse. When the tumor width reached 2.5–3 mm (at day 20), 40 mice were randomly grouped and treated (10 per group). YF, erlotinib, and vehicle were administered via oral gavage with continuous once-daily dosing. Tumor size was measured every 5 days for 30 days using a Vernier caliper, and the tumor weights (mg) were calculated according to a standard formula: [length (mm) × width (mm)^2^]/2. At the end of treatment, mice were euthanized with deep anesthesia, and tumor samples were collected for gross analyses.

### 2.6. Western Blotting Analysis

Tumor samples were examined by western blotting to investigate the expression of the main proteins in PI3K/Akt pathway. Levels of Akt1, p-Akt1 (pT308, pS473), PTEN, and EGFR were investigated in the tumor tissue homogenates.

Tumor tissue from each mouse was homogenized (PowerGen 125; Fisher Scientific, Hampton, NH, USA) in cell lysis buffer (Beyotime, Jiangsu, China) supplemented with protease inhibitors and Phosphatase Inhibitor Cocktails 1 and 2 (Sigma). Protein extraction and Western blotting assays were performed as previously described [14].

### 2.7. Reagents and Antibodies

Rabbit polyclonal antibody against epidermal growth factor receptor (EGFR) and antibodies against phospho-EGFR (pY1068), Akt1, phospho-Akt1 (pS473), phospho-Akt1 (pT308), and PTEN (all from Epitomics-Abcam Co., Burlingame, CA, USA) and a mouse monoclonal antibody against GAPDH (Abcam) were used.

### 2.8. Immunohistochemistry

Based on the specific binding of antigen and antibody, immunohistochemistry was performed to detect the expression level and spatial distribution of EGFR, PTEN, and Akt1. After treatment for 30 days, the mice were euthanized by deep anesthesia, and the xenograft tumor tissues were fixed overnight in 4% paraformaldehyde and then dehydrated and embedded in paraffin wax. The wax blocks were sectioned at 4 *μ*m, followed by immunohistochemical staining using the Envision two-step method, in accordance with the manufacturer's instructions. Cytoplasmic expression of PTEN, EGFR, and Akt1 proteins was observed under light microscopy.

### 2.9. Real-Time Quantitative PCR

To investigate EGFR, Akt1, and PTEN, real-time quantitative PCR (qPCR) was used. Total RNA was extracted and reverse-transcribed into cDNA with reverse transcription using avian myeloblastosis virus transcriptase (D2620; TaKaRa). Polymerase chain reaction (PCR) was performed with ExTaq (DRR041A; TaKaRa). Primer sequences are listed in Table 1.

### 2.10. Statistical Analysis

The experimental data discussed in the text were expressed as mean ± standard deviation. One-way analysis of variance and* post hoc* Tukey's honest significant difference test for multiple comparisons were performed. The statistical software used was SPSS (version 11.0; SPSS Inc., Chicago, IL, USA). Significance level was defined as *P* < 0.05.

## 3. Results

### 3.1. HPLC Analysis of YF

**Figure 1:**
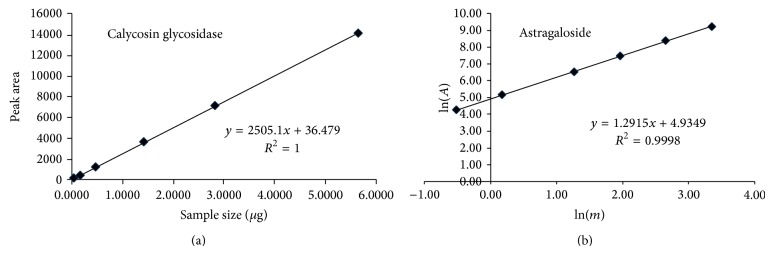
Standard curves for calycosin glycosidase (a) and astragaloside (b).

Pachymic acid was not detected by HPLC, indicating that calycosin glycosidase and astragaloside were the main compounds of YF (Table 2, Figure 1).

### 3.2. Inhibition of Tumor Growth

**Figure 2:**
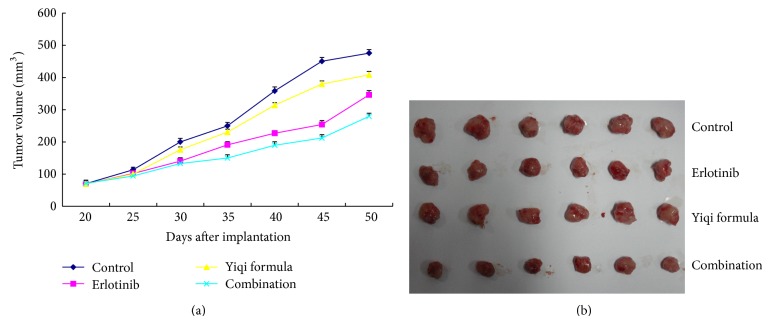
(a) Tumor growth (volume) with time. (b) The selected tumor samples of each group from mice on day 50.

All tumor grew over time. At the end of treatment, the average volume of each group was as follows: control group > YF > erlotinib > YF-erlotinib combination. The tumor weight was lower in all three treatment groups lower than in the control group (*P* < 0.05, *P* < 0.01), and there was no statistical difference between the YF and erlotinib groups (*P* > 0.05). The tumor weight of the combination treatment was lower than that of erlotinib (Figure 2, Table 3).

### 3.3. Expression of Signaling Proteins in the PI3K/Akt Pathway

**Figure 3:**
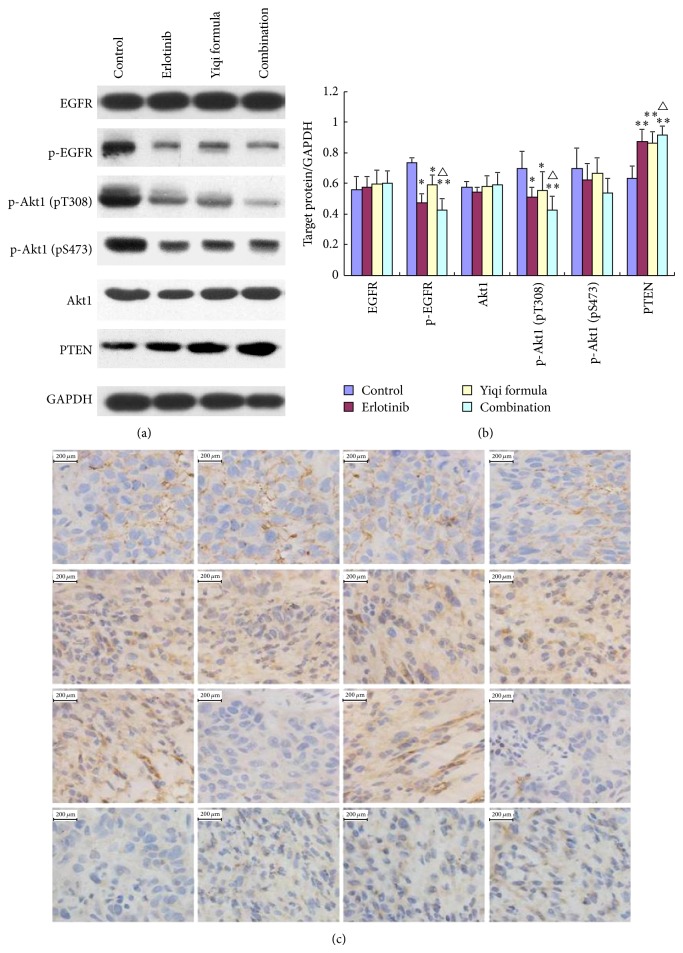
(a) Expression levels of EGFR, p-EGFR, p-Akt1 (pT308), p-Akt1 (pS473), Akt1, and PTEN in TNBC xenografts. (b) The ratio of target protein and GAPDH. ^*^ *P* < 0.05 and ^**^ *P* < 0.01 compared with control. ^△^ *P* < 0.05 compared with erlotinib. (c) Immunohistochemistry of EGFR, Akt1, p-Akt1 (pT308), and PTEN in TNBC xenografts. Nuclei were stained blue by DABI (3,3N-diaminobenzidine tetrahydrochloride).

Western blots showed expression of Akt1, p-Akt1 (pT308, pS473), PTEN, and EGFR in the tumor tissue homogenates (Figure 3(a)).

EGFR and Akt1 are transmembrane proteins, and there was no difference between the four groups for these two proteins. Immunohistochemistry was used to identify the expression level and spatial distribution of EGFR, PTEN, and Akt1 expression (Figure 3(c)). Erlotinib downregulated p-EGFR and p-Akt1 (pT308) and upregulated PTEN compared with control (*P* < 0.05, *P* < 0.01). YF reduced expression of p-EGFR, p-Akt1 (pt308) (*P* < 0.05), and increased expression of PTEN compared with control (*P* < 0.01). Combination group showed better efficiency compared with control, erlotinib (*P* < 0.05). Immunostaining gave similar results.

### 3.4. Expression of PTEN, EGFR, and Akt1 mRNA in the PI3K/Akt Pathway

**Figure 4:**
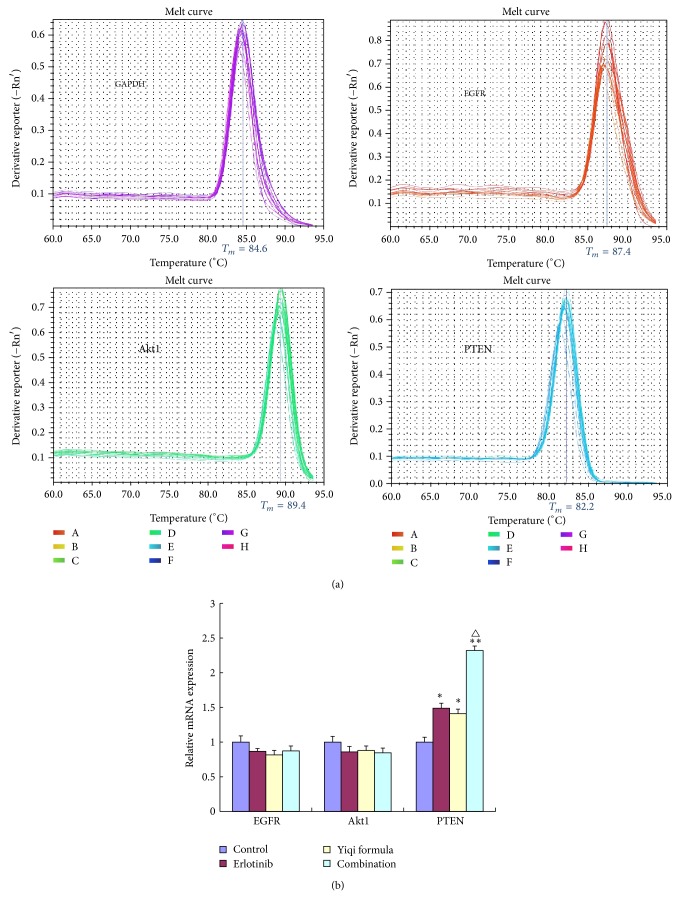
(a) Melting curves of EGFR, Akt1, and PTEN. (b) Relative mRNA expression of EGFR, Akt1, and PTEN. ^*^ *P* < 0.05 and ^**^ *P* < 0.01 compared with control. ^△^ *P* < 0.05 compared with erlotinib.

The expression level of EGFR and Akt1 mRNA did not change with the YF-erlotinib combination compared with the control (*P* > 0.05). The PI3K/Akt pathway is negatively regulated by the tumor-suppressor gene* PTEN*, which is localized to chromosome 10. The mRNA expression level of PTEN was increased in the three treatment groups compared with control (*P* < 0.05), and the YF-erlotinib combination shows better efficacy compared with erlotinib alone (*P* < 0.05) (Figure 4).

## 4. Discussion

Gene expression profiling of BCs has identified specific subtypes with important clinical, biological, and therapeutic implications. In China, TCM practitioners are using a variety of techniques in an effort to treat diseases; TCM drugs have potential synergistic therapeutic effects with gene therapy in cancer treatments [15]. Currently, BC is categorized into luminal A, luminal B, Her-2-overexpressing, triple-negative, and normal subtypes. TNBC is an aggressive subtype that has a poor prognosis. TNBC is hormone receptor-negative and Her-2/neu-negative and so is not responsive to hormone therapy or the anti-Her-2 monoclonal antibody trastuzumab; thus therapy options for TNBC are restricted to conventional systemic cytotoxic chemotherapies. Although patients with TNBC have higher response rates to chemotherapy, they show a higher rate of recurrence and metastasis than patients with other types of BC. No approved systemic therapies are available to treat patients with TNBCs once they are refractory to conventional cytotoxic chemotherapy, and it is unclear whether therapeutic targets are available for the various TNBCs. EGFR is a transmembrane receptor tyrosine kinase (RTK) of the ERBB/HER family, which includes EGFR/Her-1, ERBB2/Her-2, ERBB3, and ERBB4 [16, 17]. Like many other RTKs, EGFR has important roles in proliferation and differentiation of normal cells and in malignant transformation. EGFR/Her-1 is overexpressed in up to 70% of patients with TNBC and has an important role in proliferation, migration, and protection against apoptosis [18, 19]. This marker is a predictor of poor prognosis and is associated with menopausal status, lymph node status, P53 expression, Ki-67 expression, and lymphatic vascular invasion [20]. To date, clinical trials for TNBCs using EGFR inhibitors (EGFRi) as single agents have yielded disappointing results [21–23]. In China, many TCM herbs (TCMH) have been widely used in combination with EGFR-TKI, in order to minimize the toxicity and maximize the curative effect of the therapy. A systematic review and meta-analysis have found TCMH intervention can increase efficacy and reduce toxicity when combined with EGFR-TKI for advanced non-small-cell lung cancer (NSCLC) [24]. Some authors have reported that combinational treatment using EGFRi, such as gefitinib or erlotinib with PI3K/Akt pathway inhibitors (PI3K/AKTi), demonstrates a synergistic, antiproliferative effect in TNBC cell lines [25, 26]. These results suggest that pharmacological inhibition of EGFR by combinations of PI3K/AKTi is a potential therapeutic approach to treat TNBC.

The PI3K/Akt pathway is known to be downstream of EGFR activation, and the network plays a key regulatory function in the survival, proliferation, migration, metabolism, angiogenesis, and apoptosis of cells [27]. Activation of the oncogenes* PIK3CA *and* Akt* and loss of* PTEN* are commonly found in BC. More than 70% of breast tumors have molecular alterations in at least one component of the pathway [28]. TNBC has also shown enhanced PI3K pathway activity, mainly through PTEN loss [29]. The loss of the tumor suppressor gene* PTEN* has been reported in approximately 44.3% of TNBCs and may play a major role in the pathogenesis of these tumors and the poor clinical outcomes of the patients [30].

TCM has been widely applied for cancer care in China. As complementary medicine adjuvant or postal to conventional treatment, it plays an important role in minimizing disability, protecting cancer patients against suffering from complications, and helping patients to live well [31]. In China, most postoperative patients with BC receive TCM treatment to relieve symptoms, improve quality of life, and prolong survival. Yiqi is the main treatment principle for increasing the overall response rate and is widely used in the treatment of TNBC; therefore we have performed a series of studies on this principle. In our previous work [10–13], we found that injections of different concentrations of* Astragalus* induced apoptosis of TNBC cell lines and down-regulated expression of EGFR and p53 proteins. Further work showed that injection of* Astragalus* and its main ingredients inhibited TNBC cell proliferation and downregulated p-Akt1. These results indicate that* Astragalus* regulates p-Akt1 and PTEN in the PI3K/Akt pathway. To further our research, we selected YF, based on the Yiqi Fuzheng principle of TCM on TNBC and our previous clinical experience, and investigated its effect and related mechanisms* in vivo*. Using orthogonal test design, the best proportion of* Astragalus* and* P. cocos* (24.5 g : 14.5 g) was obtained through a number of* in vitro* experiments in our research team. HLPC was used to identify the main compounds of YF. We hypothesized that the effect of YF on TNBC would be associated with the PI3K pathway.

EGFRi as single agents have turned out to be disappointing in patients with TNBC. Inhibition of EGFR and the PI3K/Akt pathway shows a synergistic effect in TNBC cells. Because our previous study suggested that* Astragalus* had an effect on the PI3K/Akt pathway, we hypothesized that YF, which includes* Astragalus* as one of its active components, might enhance the antitumor effect of EGFRi such as erlotinib. In our study, the inhibition rate of tumor weight after erlotinib treatment on TNBC xenografts was 26.47%, consistent with the literature reports [32]. When erlotinib was used in combination with YF, this inhibition rate was increased to 39.15%. This result suggests that the combination of YF and erlotinib results in enhanced inhibition of TNBC* in vivo* and that YF improves the efficiency of erlotinib.

Dean et al. found that PTEN loss occurred in 48.3% of cases of TNBC and was significantly associated with younger age at diagnosis (47 years compared with 57 years in those without PTEN loss; *P* = 0.005); independent predictors of PTEN loss were late stage at presentation (*P* = 0.026). The study also found pAKT expression (92%) with concomitant loss of PTEN expression in the majority of TNBC cases (63%) [33]. In recent studies, there has been consensus that a high percentage of PTEN loss and presence of p-Akt1 in TNBC indicates poor disease-free survival [34]. These results indicate PTEN and p-Akt1 as potential targets in the treatment of TNBC.

We examined the effects of the erlotinib-YF combination on the PI3K/Akt signaling pathway. At the protein level, erlotinib, YF, and the erlotinib-YF combination reduced p-Akt1 and p-EGFR level and increased PTEN level compared with the control (*P* < 0.05 and *P* < 0.01, resp.). The combination showed better efficacy compared with erlotinib alone (*P* < 0.05).

We used immunohistochemistry staining to investigate the spatial distribution of EGFR, PTEN, Akt1, and p-Akt1 (pT308). This also showed that the YF-erlotinib combination was more effective in increasing PTEN and reducing p-Akt1 (pT308), consistent with the western blotting results. We assessed the mRNA levels of PTEN, Akt1, and EGFR and found that the three treatment groups had an increased level of PTEN mRNA compared with the control (*P* < 0.01), and the YF-erlotinib combination showed better efficacy compared with erlotinib (*P* < 0.05). These results indicate that the effects of YF on TNBC are mainly through regulating the PI3K/Akt pathway, especially downregulating p-EGFR and p-Akt1 (pT308) and upregulating PTEN.

In conclusion, YF enhances the effect of erlotinib for treatment of TNBC xenografts, with the drugs having a synergistic effect, and regulating PTEN and p-Akt1 in the PI3K/Akt pathway. This study suggests that YF is a promising agent for the treatment of TNBC and that the Yiqi principle is an exciting adjunct to systemic therapy for TNBC that should be considered for testing in clinical trials.

## Figures and Tables

**Table 1:** Primers used for detecting the gene expression by reverse real-time PCR.

Gene	Primer sequence 5′ → 3′	
	Forward primer	Reverse primer
*EGFR *	GGACGACGTGGTGGATGCCG	GGCGCCTGTGGGGTCTGAGC
*Akt *	ATGAGCGACGTGGCTATTGT	GAGGCCGTCAGCCACAGTCT
*PTEN *	TCACCAACTGAAGTGGCTAAAGA	CTCCATTCCCCTAACCCGA
*GAPDH *	GAAGGTGAAGGTCGGAGTC	GAAGATGGTGATGGGATTTC

**Table 2:** Results of Yiqi formula assay.

Component	Quantity, *μ*g/mL
Calycosin glycosidase	147.97
Astragaloside	95.5
Pachymic acid	0.508

**Table 3:** Degree of tumor inhibition.

Group	Number	Tumor weight, mg	Inhibition, %
Control	10	513.73 ± 46.23	—
Erlotinib	10	377.80 ± 61.29∗	26.47
Yiqi formula	10	425.20 ± 52.14∗	17.24
Combination	10	312.67 ± 36.98^∗∗△^	39.15

^*^ *P < *0.05, ^**^ *P < *0.01, compared with control. ^△^ *P < *0.05, compared with erlotinib.
